# Mogamulizumab, an anti-CCR4 monoclonal antibody, is a potent therapeutic option for adult T-cell leukemia-lymphoma

**DOI:** 10.1186/1742-4690-11-S1-O3

**Published:** 2014-01-07

**Authors:** Atae Utsunomiya

**Affiliations:** 1 Department of Hematology, Imamura Bun-in Hospital, Kagoshima City, Kagoshima, Japan

## 

Adult T-cell leukemia-lymphoma (ATL) is a rare and aggressive T-cell malignancy associated with human T-cell lymphotropic virus type 1 (HTLV-1). ATL remains an extremely difficult disease to treat, and therefore, the development of novel therapies is highly needed. Because CC chemokine receptor 4 (CCR4) is frequently over-expressed on ATL cells from patients (Clin Cancer Res. 2003;9:3625), mogamulizumab, a defucosylated humanized anti-CCR4 antibody, has been developed for the treatment of ATL in Japan. In a phase I study in patients with relapsed CCR4-positive T-cell malignancies, mogamulizumab was well tolerated up to 1 mg/kg and encouraging efficacy was observed (JCO 2010;28:1591). In a subsequent pivotal phase II study in CCR4-positive relapsed ATL patients, mogamulizumab exhibited an overall response rate (ORR) of 50% including 8 complete responses (CR) (JCO 2012;30:837), leading to its approval in Japan in 2012 for relapsed/refractory ATL. Furthermore, with the aim of establishing a new standard therapy for untreated ATL, we conducted a randomized phase II study of VCAP-AMP-VECP, a dose-intensified multi-agent chemotherapy, with or without mogamulizumab (arm-A or arm-B). The CR rate (primary endpoint) and ORR (secondary endpoint) were higher in arm-A than in arm-B (52% vs. 33% and 86% vs. 75%, respectively). Median progression-free survival was also longer in arm-A (259 days vs. 192 days). The most common treatment-related AEs were hematological toxicity in both arms. These results suggest that mogamulizumab could provide a new effective treatment option for both relapsed/refractory and untreated ATL.

## Trial registration

http://ClinicalTrials.gov NCT01173887

